# Prediction and identification of mouse cytotoxic T lymphocyte epitopes in Ebola virus glycoproteins

**DOI:** 10.1186/1743-422X-9-111

**Published:** 2012-06-13

**Authors:** Shipo Wu, Ting Yu, Xiaohong Song, Shaoqiong Yi, Lihua Hou, Wei Chen

**Affiliations:** 1 State Key Laboratory of Pathogens and Biosecurity, Beijing Institute of Microbiology and Epidemiology, No.20 Dongdajie Street, Fengtai district, Beijing, 100071, People's Republic of China

**Keywords:** Ebola virus, T cell epitope, Replication-deficient adenovirus, Computer-assisted algorithms

## Abstract

**Background:**

Ebola viruses (EBOVs) cause severe hemorrhagic fever with a high mortality rate. At present, there are no licensed vaccines or efficient therapies to combat EBOV infection. Previous studies have shown that both humoral and cellular immune responses are crucial for controlling Ebola infection. CD8^+^ T cells play an important role in mediating vaccine-induced protective immunity. The objective of this study was to identify H-2^d^-specific T cell epitopes in EBOV glycoproteins (GPs).

**Results:**

Computer-assisted algorithms were used to predict H-2^d^-specific T cell epitopes in two species of EBOV (*Sudan* and *Zaire*) GP. The predicted peptides were synthesized and identified in BALB/c mice immunized with replication-deficient adenovirus vectors expressing the EBOV GP. Enzyme-linked immunospot assays and intracellular cytokine staining showed that the peptides RPHTPQFLF (*Sudan* EBOV), GPCAGDFAF and LYDRLASTV (*Zaire* EBOV) could stimulate splenoctyes in immunized mice to produce large amounts of interferon-gamma.

**Conclusion:**

Three peptides within the GPs of two EBOV strains were identified as T cell epitopes. The identification of these epitopes should facilitate the evaluation of vaccines based on the Ebola virus glycoprotein in a BALB/c mouse model.

## Background

Ebola viruses (EBOVs) are enveloped, non-segmented, negative-strand RNA viruses belonging to the family *Filoviridae*. They are known to cause lethal hemorrhagic fever in humans and non-human primates with a mortality rate up to 90% [1,2]. EBOVs transmit among human and nonhuman primate populations through contact with infected blood, bodily fluids or tissues; moreover, the intentional release of EBOVs would probably result in mucosal infection by small-particle aerosol dispersion [3-5]. Although a considerable worldwide threat exists should EBOVs spread globally, currently, there are no licensed vaccines or effective treatments. Five different species of EBOV have been identified: *Zaire* (ZEBOV); *Sudan* (SEBOV); *Ivory Coast*; *Reston*; and the newly discovered *Bundibugyo*[6]. Among these species, infections with SEBOV and ZEBOV are the most commonly occurring and have caused the greatest number of deaths. The EBOV envelope glycoprotein (EBOV-GP) forms spikes on the surface of mature virions, and has been shown to be an effective target for vaccine design [7]. Several vaccine candidates based on the EBOV-GP have been shown to protect non-human primates: Ebola virus-like particles [8,9]; a replication-deficient adenovirus expressing the EBOV-GP [10-13]; a replication-competent vesicular stomatitis virus expressing EBOV-GP [14-16]; and a recombinant paramyxovirus expressing EBOV-GP [17]. Although humoral responses to EBOV are very important, EBOV-specific CD8^+^ cytotoxic T lymphocytes (CTLs) are necessary for viral control and clearance (reviewed in [18]).

CTL epitopes are increasingly important as research targets for the development of vaccines and immunotherapies, and are also very useful for evaluating the efficacy of vaccines. Experimental methods for identifying CTL epitopes involve multiple overlapping peptides spanning individual antigens, as well as complete viral proteomes. These methods are expensive and time-consuming. Computational prediction methods minimize the number of validation experiments, and significantly speed up the process of epitope prediction [19]. There are many epitope prediction programs now available on the internet, and computational prediction of CTL epitopes has become a topic of vigorous research and development activity.

For EBOVs, mouse model represents the necessary first step in the development of a potential vaccine candidate that can then be further tested in primates and humans [20,21]. Identification of CD8^+^ T cell epitopes in EBOV antigens presented by murine major histocompatability complex (MHC) molecules is essential to prove the effectiveness of any vaccination strategy in this animal model. In this study, the H-2^d^-specific T cell epitopes in the envelope glycoprotein (GP) of SEBOV and ZEBOV were predicted using software. The predicted peptides were synthesized and identified by intracellular cytokine assays using splenocytes from rAd-EBOV-GP (replication-deficient adenovirus vectors expressing the EBOV GP)-vaccinated mice.

## Results

### Computational prediction of EBOV-GP CTL epitopes

The prediction of class I MHC-restricted epitopes of SEBOV-GP and ZEBOV-GP sequences for the K, D, and L loci of the mouse haplotype H-2^d^ (BALB/c) was performed using prediction programs available on the internet (Table 1). For each program, the peptides that ranked among the top five prediction results were selected. All selected peptides were rearranged according to frequency of occurrence in the top five ranking for all the prediction programs. Twelve peptides were selected for further evaluation (Table 2). Among those peptides, LYDRLASTV (LV, H-2K^d^) and EYLFEVDNL (EL, H-2K^d^) were previously reported as CTL epitopes in ZEBOV-GP [21].

**Table 1:** Programs for MHC class I epitope prediction used in this study

**Programs**	**URL**
BIMAS	http://www-bimas.cit.nih.gov/molbio/hla_bind/
IEDB (SMM)	http://tools.immuneepitope.org/analyze/html/mhc_binding.html
NetMHC	http://www.cbs.dtu.dk/services/NetMHC/
NetCTLpan	http://www.cbs.dtu.dk/services/NetCTLpan/
nHLAPred (Compred)	http://www.imtech.res.in/raghava/nhlapred/comp.html
PREDEP	http://margalit.huji.ac.il/Teppred/mhc-bind/index.html
ProPred-I	http://www.imtech.res.in/raghava/propred1/
SYFPEITHI	http://www.syfpeithi.de/Scripts/MHCServer.dll/EpitopePrediction.htm

**Table 2:** **Amino acid sequence of the predicted SEBOV-GP and ZEBOV-GP CD8**^**+**^**T cell epitopes**

**Ebola virus**	**peptides**		
**Subtypes**	**H-2K**^**d**^	**H-2D**^**d**^	**H-2L**^**d**^
*Zaire Ebola virus*	LYDRLASTV (LV)	QGPTQQLKT (QT)	GPCAGDFAF (GF)
	EYLFEVDNL (EL)	KKPDGSECL (KL)	LPQAKKDFF (LF)
*Sudan Ebola virus*	LYDRLASTV (LV)	TGPCDGDYA (TA)	RPHTPQFLF (RF)
	SFFVWVIIL (SL-9)	KKPDGSECL (KL)	SYYATSYL (SL-8)

### Identification of H-2^d^-restricted CD8^+^ T cell epitopes in SEBOV-GP

**Figure 1:**
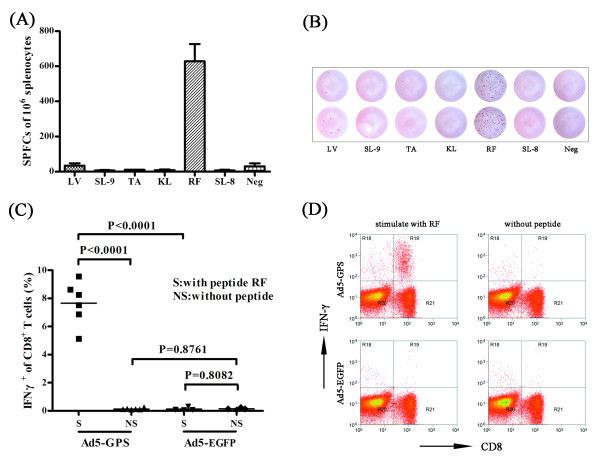
** Identification of H-2**^**d**^**-restricted epitopes in SEBOV-GP.(A, B)** BALB/c mice were immunized with Ad5-GPS twice, at an interval of 4 weeks. Splenocytes were harvested 10 days after the second immunization and re-stimulated *in vitro* with the predicted peptides from SEBOV-GP for use in IFN-γ ELISPOT assays. RF was observed to induce strong IFN-γ-specific spot forming. A negative control without peptide was included. **(C, D)** To further confirm the peptide identified in the ELISPOT assay, BALB/c mice were immunized with Ad5-GPS or Ad5-EGFP (as a control) twice, splenocytes were re-stimulated *in vitro* with RF or without peptides and the responding CD8^+^ T cells were visualized by intracellular IFN-γ staining.

The rAd-EBOV-GP was observed to induce a robust CD8^+^ T cell response and offers protection against lethal Ebola virus challenge [12]. To identify H-2^d^ specific CD8^+^ T cell epitopes in SEBOV-GP, a double injection of a recombinant replication-deficient adenovirus serotype 5 expressing the glycoprotein of SEBOV (Ad5-GPS) was performed to obtain a strong cellular immune response. Splenocytes were harvested 10 days after the second immunization and re-stimulated *in vitro* with the predicted peptides in an interferon (IFN)-γ enzyme-linked immunospot (ELISPOT) assay (Figure 1A, B). Of the six predicted peptides, only RPHTPQFLF (RF) showed specific stimulation of an IFN-γ response. LV, which was identified as the H-2^d^-specific epitope in ZEBOV-GP [22], showed no response.

To further confirm RF was a specific CTL CD8^+^ epitope, BALB/c mice were immunized with Ad5-GPS or control (Ad5-EGFP) twice with an interval of 4 weeks. At 10 days after the second immunization, splenocytes were re-stimulated *in vitro* with RF or without peptides, in the presence of brefeldin A for 6 h. This was followed by intracellular IFN-γ staining and flow cytometry analysis (Figure 1C, D). It was shown that RF could stimulate splenocytes from mice immunized with Ad5-GPS, resulting in a robust IFN-γ response in CD8^+^ T cells. The splenocytes from mice immunized with control, or cells without stimulation, demonstrated no response. RF represents the first H-2^d^-restricted peptide described in SEBOV-GP.

### Identification of H-2^d^-restricted CD8^+^ T cell epitopes in ZEBOV-GP

**Figure 2:**
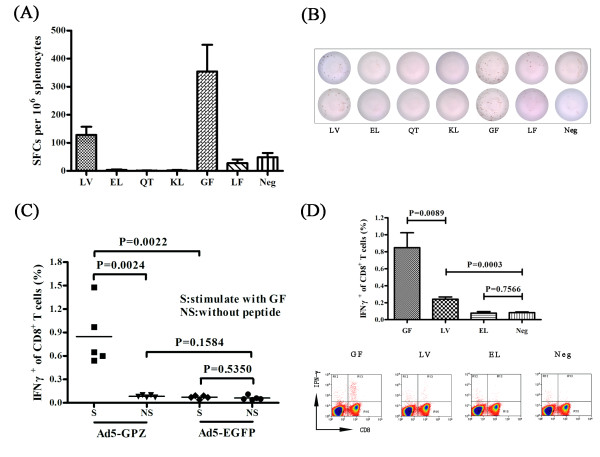
** Identification of H-2**^**d**^**-restricted epitopes in ZEBOV-GP.(A, B)** BALB/c mice were immunized with Ad5-GPZ twice, at an interval of 4 weeks. Splenocytes were harvested 10 days after the second immunization and re-stimulated *in vitro* with the predicted peptides from ZEBOV-GP for use in the IFN-γ ELISPOT assay. GF and LV were observed to induce IFN-γ-specific spot forming. A negative control without peptide was included. **(C, D**) To further confirm the peptides identified in the ELISPOT assay and to compare the stimulation effect of GF, LV and EL, BALB/c mice were immunized with Ad5-GPZ or Ad5-EGFP (as a control) twice. Splenocytes were re-stimulated *in vitro* with GF, LV, EL or without peptides and the responding CD8^+^ T cells were visualized by intracellular IFN-γ staining.

Using the same strategy, two H-2^d^-restricted epitopes were identified in ZEBOV-GP (Figure 2). BALB/c mice were immunized twice with a recombinant replication-deficient adenovirus serotype 5 expressing the glycoprotein of ZEBOV (Ad5-GPZ), splenocytes were re-stimulated with the predicted peptides, and positive IFN-γ T cell responses were noted with LV and GPCAGDFAF (GF) (Figure 2A, B). LV, a CTL epitope of ZEBOV-GP specific to H-2K^d^ had been identified previously [22]. However, GF represented a novel epitope in ZEBOV-GP. EL, which was identified as a H-2K^d^-specific epitope in ZEBOV-GP [22] did not result in an IFN-γ response from T cells in this study. Further intracellular IFN-γ staining confirmed that immunization with Ad5-GPZ was sufficient to stimulate a strong CD8^+^ T cell response against GF, whereas control Ad5-EGFP did not (Figure 2C). At the same time, GF was more efficient at inducing IFN-γ secretion than LV (Figure 2D), suggesting that GF represented a more sensitive epitope for the detection of IFN-γ, especially when the level of IFN-γ secretion was low.

## Discussion

In this study, we identified H-2^d^-restricted CD8^+^ T cell epitopes in SEBOV-GP and ZEBOV-GP. The CD8^+^ T cell epitopes in SEBOV-GP and ZEBOV-GP were predicted using internet-based prediction programs. The peptides with the highest scores and greatest frequency in the top five rankings for all programs were selected, with two peptides for each loci (D, K and L) of the mouse haplotype H-2^d^ identified.

Three peptides, RF (H-2L^d^) in SEBOV-GP, along with LV (H-2K^d^) and GF (H-2L^d^) in ZEBOV-GP, out of 12 predicted peptides were able to induce strong IFN-γ responses. RF and GF are newly identified epitopes in SEBOV-GP and ZEBOV-GP, respectively. LV appeared in the predicted CTL epitopes for both SEBOV-GP and ZEBOV-GP, and was previously identified as an effective epitope in ZEBOV-GP [22]. However LV could not induce IFN-γ secretion from the splenocytes of Ad5-GPS-immunized mice in this study. The amino acid homology between SEBOV-GP and ZEBOV-GP was 70.1%, and we are uncertain as to why there were differences between these proteins. EL, another peptide previously reported as a CTL epitope in ZEBOV-GP [22], was not effective in our study, a result similar to that seen in a previous study using DNA as the vaccination platform [23].

Intracellular cytokine staining and ELISPOT assays are the most commonly used quantitative assays for counting peptide-specific T cells. ELISPOT is a key methodology used in the identification of novel epitopes because of its high throughput. However, intracellular cytokine staining has the added advantage that qualitative analysis can be performed, making it possible to identify specific cell subpopulations that contribute to cytokine production. Wells that were positive for the primary ELISPOT assay can be retested by intracellular cytokine staining to determine if cytokine-secreting cells are CD4^+^ or CD8^+^. The intracellular cytokine staining results showed that RF and GF are EBOV-GP specific and induced robust IFN-γ responses in CD8^+^ T cells, demonstrating that they are the novel CTL epitopes. Interestingly, the two novel CTL epitopes were H-2L^d^ restricted. Intracellular IFN-γ staining in splenocytes of Ad5-GPZ-immunized mice showed that GF was able to induce stronger development of a peptide-specific IFN-γ response in CD8^+^ T cells, suggesting that GF represents a more efficient CD8^+^ CTL epitope in ZEBOV-GP.

*In silico* methods together with *in vivo/in vitro* validation have proven to be a quick and effective strategy for identifying T cell epitopes [24-26]. Compared with the expensive and time-consuming overlapping peptides method, this strategy not only reduces the workload and cost, but also improves the rate of test, and has become the most popular approach for CTL epitope research. To predict a novel epitope, programs based on proteasomal cleavage, TAP transport, MHC binding or a combination of these have been developed. However, compared with highly selective peptide binding to MHC molecules, proteasomal cleavage and TAP transport have little influence on epitope generation [27]. There is significant interest in the development of computational methods for predicting the binding capability of peptides to MHC molecules as a first step towards selecting peptides for actual screening. In this study, MHC binding-based algorithms, integrated methods, as well as structure-based algorithms were used. To compare the predictive accuracy of the programs in this study, the ranking of the identified peptides in each program were summarized (Table 3). NetMHC, a prediction program based on artificial neural networks (ANNs) and position-specific scoring matrices (PSSMs), seemed to provide the best performance. This result corresponded with previous comparison tests [28,29]. Other prediction programs, such as IEDB (SMM), NetCTLpan, SYFPEITHI, BIMAS, nHLAPred (Compred) and Propred-1 also demonstrated high predictive accuracy. IEDB (SMM) is one of the class I MHC peptide-binding prediction methods provided through the immune epitope database analysis resource that models binding specificity of an MHC molecule using PSSMs [27]. NetCTLpan is a pan-specific MHC class I epitope predictor, which integrates prediction of proteasomal cleavage, TAP transport efficiency and peptide-MHC binding [30]. SYFPEITHI, one of the first algorithms available online, is a motif-matrix-based prediction method for MHC binding prediction [31]. However, PREDEP, a structure-based algorithm, showed the lowest performance levels in our study. It would appear that sequence-based methods are computationally more efficient than structure-based methods. However, sequence-based methods do not provide a structural interpretation of results, which is of importance for designing peptide vaccines and drug-like molecules [29].

**Table 3:** Rankings of the identified peptides from the prediction programs

Programs	Epitopes	Ranking		
		LYDRLASTV (H-2K^d^)	GPCAGDFAF (H-2L^d^)	RPHTPQFLF (H-2L^d^)
BIMAS		5	2	2
IEDB (SMM)		2	1	1
NetCTLpan		2	3	1
NetMHC		1	1	1
nHLAPred (Compred)		5	3	7
PREDEP		-	93	4
Propred-1		5	2	2
SYFPEITHI		1	1	4

To date, some EBOV derived CD8^+^ T cell epitopes have been identified. Rao *et al.* identified a H-2^k^-specific and two H-2^d^-specific murine EBOV-GP CTL epitope by immunizing mice with a liposome encapsulated by irradiated EBOV [22,32]. However, one of the identified CTL epitopes (EL) showed no response in another study [23], and also in this work. Other studies have used Venezuelan equine encephalitis virus-based EBOV vaccines to identify the H-2^d^- or H-2^b^-restricted CTL epitopes in EBOV GP, NP, VP24, VP30, VP35 and VP40 [33,34]. At the same time, Simmons *et al.* attempted to map murine CTL epitopes of EBOV-NP using overlapping peptides. They identified a single H-2^d^- and two H-2^b^-restricted CTL epitopes [35]. Following on from this, a combination of computational prediction together with *in vitro*/*in vivo* validation methods were used to identify the HLA-A2.1-specific CTL epitopes in EBOV-NP [36]. However, those predictions are almost entirely based upon ZEBOV, and no effective SEBOV-GP CTL epitope has been reported. Here, we identified a H-2^d^-restricted CTL epitope in SEBOV-GP, and at the same time, a novel H-2^d^-specific epitope in ZEBOV-GP.

## Conclusions

RF and GF were the best candidates to measure CD8^+^ T cell responses in BALB/c mice model after vaccination with SEBOV or ZEBOV vaccines based on EBOV-GP. The identification of H-2^d^-restricted CD8^+^ T cell epitopes for EBOV-GP will contribute to analyzing the efficacy of different vaccination protocols based upon the GP in the BALB/c mice model. Our findings also illustrate how a hybrid immune-computational approach may be useful for biologists in identifying candidate epitopes.

## Methods

### Computational prediction of candidate CTL epitopes in EBOV-GP

H-2^d^ (H-2K^d^, H-2D^d^ and H-2L^d^)-restricted epitopes of SEBOV-GP and ZEBOV-GP were predicted using BIMAS, IEDB (SMM), NetMHC, NetCTLpan, nHLAPred (Compred), PREDEP, ProPred-I and SYFPEITHI (Table 1). The top five ranking peptides from each program were selected, and rearranged by frequency of occurrence in all prediction programs. Considering the scores of peptides in the prediction program and their frequency of ranking in the top five, the most likely H-2K^d^-, H-2D^d^- or H-2L^d^-specific binding peptides (two peptides for each) were selected for further evaluation.

### Peptides

All peptides for identification were synthesized by GL Biochem Ltd (Shanghai, China) and provided at >95% purity, as verified by high-performance liquid chromatography and mass spectrometry analysis. Peptides were dissolved in sterile phosphate-buffered saline (PBS), diluted to 2 mg/ml and frozen at -80 °C until required.

### Construction of the vaccines

Briefly, Ad5-GPS and Ad5-GPZ were generated using the appropriate open reading frames for the genes encoding the glycoprotein of SEBOV (Genbank accession number EVU28134) or ZEBOV (Genbank accession number NC-002549), respectively. These were cloned into the adenoviral shuttle plasmid pDC316. The shuttle plasmid and the adenoviral backbone plasmid (pBHGlox_E1, 3Cre) were co-transfected into HEK293 cells using Lipofectamine™ Reagent (Invitrogen, Carlsbad, California, USA), following the manufacturer’s instructions. Transfected cells were maintained until adenovirus-related cytopathic effects were observed. The adenoviruses were harvested and confirmed by polymerase chain reaction (PCR). Positive recombinant adenoviruses were reamplified in HEK293 cells and purified by ion exchange (SOURCE 15Q) and size exclusion. The viruses were titrated on HEK293 cells using an Adeno-X™ Rapid Titer Kit (Clontech, Japan). The resulting titers were scored as infectious units (IFU)/ml.

### Animal experiments and splenocytes dissociation

Female BALB/c (H-2^d^) mice that were 4–6-weeks-old were purchased from the Laboratory Animal Centre in National Institute for the Control of Pharmaceutical and Biological Products (P.R. China). Mice were immunized with 1 × 10^7^ IFU of adenovirus (Ad5-GPS, Ad5-GPZ, or the control adenovirus vector Ad5-EGFP) via an intramuscular route twice, at an interval of 4 weeks. All mice were handled according to protocols approved by the Laboratory Animal Care and Use Committee, of the Beijing Institute of Microbiology and Epidemiology, and conformed with national guidelines on the ethical use of laboratory animals. Splenocytes from the immunized and control mice were harvested 10 days after the second immunization. Under aseptic conditions, spleens were pushed through a 70-μm cell strainer in complete RPMI1640 medium to prepare a single cell suspension. Splenocytes were centrifuged at 500 × *g* for 5 min, the supernatant was discarded, and red blood cells removed with ACK lysing buffer (0.15 M of NH_4_Cl, 10 mM of KHCO_3_, 0.1 mM of Na_2_EDTA, pH 7.2–7.4). Cells were washed twice in complete RPMI 1640 medium, counted, and kept on ice until required.

### ELISPOT assay

A BD™ ELISPOT Mouse IFN-γ Set was used to count peptide-specific T cells. ELISPOT plates were coated overnight at 4 °C with 5 μg/ml of anti-mouse IFN-γ antibody. The antibody-coated plates were washed two times with sterile PBS and blocked with complete RPMI medium for 2 h at room temperature. After blocking, 100 μl of splenocyte suspension (2 × 10^6^ cells/ml) containing different peptides (20 μg/ml) were added to each well. A positive control [50 ng/ml phorbol myristate acetate (PMA; Sigma, Santa clara, California, USA) and 500 ng/ml ionomycin (Sigma)] and a ‘no peptide’ negative control were included in all assays. The plates were incubated for 18 h at 37 °C/5% CO_2_. Following incubation, the wells were washed twice with deionized water and three times with washing buffer (PBS containing 0.05% Tween-20). Biotinylated anti-mouse IFN-γ was added to each well at a concentration of 2 μg/ml and the plates were incubated for 2 h at room temperature. Following three washes, streptavidin-horseradish peroxidase was added to each well and the plates were incubated for 1 h at room temperature. After four washes with washing buffer and two washes with PBS, the colorimetric reactions were developed using 3-amino 9-ethylcarbazole as a substrate. Upon visualization of the spots, the reaction was stopped by rinsing in tap water. Membranes were allowed to dry overnight in the dark and then spots were counted with a BioReader® 4000 Pro-X (Bio-Sys; Germany). Results were expressed as the number of spot-forming cells (SFCs)/10^6^ splenocytes.

### Intracellular IFN-γ staining

Splenocytes were cultured at 37 °C for 6 h with 20 μg/ml synthetic peptide, or with no peptide as the background control, or with 100 ng/ml PMA and 1 μg/ml ionomycin as the positive control. For the last 4 h of culture, 10 μg/ml brefeldin A (BFA, Sigma;) was added to block the secretion of IFN-γ. Cells were stained with PerCP/Cy5.5-conjugated anti-CD3 (clone 145-2c11; Biolegend, San Diego, California, USA) and FITC conjugated anti-CD8 (clone 53-6.7; Biolegend) monoclonal antibodies, then fixed and permeabilized with Cytofix/Cytoperm (BD Biosciences, Franklin Lake, New Jersey, USA). The permeabilized cells were incubated with PE-conjugated anti-IFN-γ (clone XMG1.2; BD Bioscience), washed and resuspended in PBS. Samples were analyzed using a Beckman Coulter CyAn^™^ ADP flow cytometer and Summit software.

### Statistical analysis

Data were expressed as means ± standard errors of the means (SEM). Statistical analysis was performed with two-tailed unpaired *t* tests using Prism 5 (GraphPad Software). *P* values of less than or equal to 0.05 were considered statistically significant.

## Competing interests

The authors declare that they have no competing interests.

## Authors’ contributions

SW carried out most of the experiments and wrote the manuscript. TY and XS participated in vaccine construction. SY provided useful advice, participated in intracellular IFN-γ staining and ELISPOT assay. WC and LH were the project leaders and were involved in project design, data analysis and finalization of the manuscript. All authors read and approved the final manuscript.
